# Dementia risk reduction in the African context: Multi‐national implementation of multimodal strategies to promote healthy brain aging in Africa (the Africa‐FINGERS project)

**DOI:** 10.1002/alz.14344

**Published:** 2024-11-07

**Authors:** Chinedu T. Udeh‐Momoh, Rachel Maina, Udunna C. Anazodo, Rufus Akinyemi, Lukoye Atwoli, Laura Baker, Darina Bassil, Karen Blackmon, Edna Bosire, Gloria Chemutai, Lucia Crivelli, Laz U. Eze, Agustin Ibanez, Dimitra Kafetsouli, Thomas K. Karikari, Linda Khakali, Manasi Kumar, Imre Lengyel, Celeste A. de Jager Loots, Francesca Mangialasche, Sylvia Mbugua, Zul Merali, Michelle Mielke, Cyprian Mostert, Eunice Muthoni, Olivera Nesic‐Taylor, Anthony Ngugi, Samuel Nguku, Adesola Ogunniyi, Adedoyin Ogunyemi, Ozioma C. Okonkwo, Njideka Okubadejo, Robert Perneczky, Tunde Peto, Roselyter M. Riang'a, Mansoor Saleh, Shaheen Sayed, Jasmit Shah, Sheena Shah, Alina Solomon, Thomas Thesen, Dominic Trepel, Valentine Ucheagwu, Victor Valcour, Sheila Waa, Tamlyn Watermeyer, Jennifer Yokoyama, Henrik Zetterberg, Miia Kivipelto, Africa‐FINGERS Study Team

**Affiliations:** ^1^ Brain and Mind Institute Aga Khan University Nairobi Kenya; ^2^ School of Public Health Sciences Wake Forest University School of Medicine Winston Salem North Carolina USA; ^3^ Division of Clinical Geriatrics Center for Alzheimer Research Karolinska Institutet Stockholm Sweden; ^4^ FINGERS Brain Health Institute Stockholm Sweden; ^5^ Global Brain Health Institute (GBHI) UCSF San Francisco California USA; ^6^ Trinity College Dublin Dublin Ireland; ^7^ Sheffield Institute for Translational Neuroscience (SITraN) University of Sheffield Sheffield UK; ^8^ Department of Neurology and Neurosurgery Montreal Neurological Institute McGill University Montreal Quebec Canada; ^9^ Medical Artificial Intelligence Laboratory Crestview Radiology, Ltd. Lagos Nigeria; ^10^ Department of Medicine & Clinical & Radiation Oncology University of Cape Town Cape Town South Africa; ^11^ Institute for Advanced Medical Research and Training College of Medicine University of Ibadan Ibadan Nigeria; ^12^ Department of Medicine Medical College East Africa the Aga Khan University Nairobi Kenya; ^13^ Harvard Center for Population and Development Studies Harvard University Cambridge Massachusetts USA; ^14^ Department of Cognitive Neurology Fleni Buenos Aires Argentina; ^15^ Trinity College Institute for Neuroscience Trinity College Dublin Dublin Ireland; ^16^ Ageing Epidemiology Research Unit School of Public Health Imperial College London London UK; ^17^ Department of Psychiatry School of Medicine University of Pittsburgh Pittsburgh Pennsylvania USA; ^18^ Grossman School of Medicine New York University New York New York USA; ^19^ School of Medicine Dentistry and Biomedical Sciences Queen's University Belfast Belfast UK; ^20^ Department of Population Health Aga Khan University Nairobi Kenya; ^21^ Tilman J. Fertitta Family College of Medicine Houston Texas USA; ^22^ Department of Radiology Aga Khan University Hospital Nairobi Kenya; ^23^ Department of Community Health and Primary Care University of Lagos Lagos Nigeria; ^24^ Division of Geriatrics and Gerontology Department of Medicine University of Wisconsin Madison Wisconsin USA; ^25^ Neurology Unit Department of Medicine College of Medicine University of Lagos Lagos Nigeria; ^26^ Ludwig Maximilien University Munich Germany; ^27^ Department of Psychiatry and Psychotherapy LMU Hospital Ludwig Maximilians Universität Munich Munich Germany; ^28^ German Center for Neurodegenerative Diseases (DZNE) Munich Munich Germany; ^29^ Munich Cluster for Systems Neurology (SyNergy) Munich Germany; ^30^ Cancer Research Unit Aga Khan University Nairobi Kenya; ^31^ Department of Pathology Aga Khan University Nairobi Kenya; ^32^ Department of Ophthalmology Aga Khan University Nairobi Kenya; ^33^ Department of Neurology Institute of Clinical Medicine University of Eastern Finland Kuopio Finland; ^34^ Department of Medical Education Geisel School of Medicine at Dartmouth Dublin Ireland; ^35^ Department of Psychology Nnamdi Azikiwe University Awka Anambra Nigeria; ^36^ University of California, San Francisco San Francisco California USA; ^37^ Centre for Dementia Prevention Clinical Brain Sciences The University of Edinburgh Edinburg UK; ^38^ Department of Psychiatry and Neurochemistry Institute of Neuroscience and Physiology the Sahlgrenska Academy at the University of Gothenburg Mölndal Sweden; ^39^ Clinical Neurochemistry Laboratory Sahlgrenska University Hospital Mölndal Sweden; ^40^ Department of Neurodegenerative Disease UCL Institute of Neurology Queen Square London UK; ^41^ UK Dementia Research Institute at UCL London UK; ^42^ Hong Kong Center for Neurodegenerative Diseases Hong Kong China; ^43^ Wisconsin Alzheimer's Disease Research Center University of Wisconsin School of Medicine and Public Health University of Wisconsin‐Madison Madison Wisconsin USA; ^44^ Institute of Public Health and Clinical Nutrition University of Eastern Finland Kuopio Finland

**Keywords:** Alzheimer's disease, brain banking, community‐based participatory research, dementia prevention trials, fluid and neuroimaging biomarkers, health economics, implementation science, retinal imaging

## Abstract

Dementia prevention in Africa is critically underexplored, despite the continent's high prevalence of modifiable risk factors. With a predominantly young and middle‐aged population, Africa presents a prime opportunity to implement evidence‐based strategies that could significantly reduce future dementia cases and mitigate its economic impact. The multinational Africa‐FINGERS program offers an innovative solution, pioneering culturally sensitive, multidomain interventions tailored to the unique challenges of the region.  Leveraging insights from landmark global studies such as Worldwide‐FINGERS and Alzheimer's Disease Neuroimaging Initiative, the program employs a multideterminant precision prevention framework, grounded in community based systems dynamics. Africa‐FINGERS further integrates cutting‐edge state‐of‐the‐art multimodal biomarker evaluations tailored to regional contexts, with the goal of advancing brain health and establishing a global standard for dementia prevention. This groundbreaking initiative highlights the potential for scalableand sustainable interventions, thus is poised to transform dementia risk reduction efforts across the continent.

**Highlights:**

- Dementia rates are escalating in Africa, largely due to longer life spans and increased prevalence of modifiable risk factors. Yet, few regional interventions have directly targeted lifestyle factors to reduce dementia risk.
- The multinational Africa‐FINGERS study will address this gap by adapting the successful FINGERS lifestyle intervention to African populations.
- Africa‐FINGERS will pioneer a culturally informed, multidomain dementia risk reduction intervention in the African region through feasibility dementia prevention trials in rural and urban sites across Kenya and Nigeria in the first instance, enrolling 600 at‐risk adults (≥ 50 years). The program adopts participatory research methods to develop culturally appropriate interventions and build infrastructure to evaluate dementia biomarkers from *ante* and *post mortem* samples. A cost‐effectiveness analysis will be conducted to guide the strategic implementation of Africa‐FINGERS into regional health systems.
- The Africa‐FINGERS strategy aligns with the Worldwide‐FINGERS framework and integrates the global Alzheimer's Disease Neuroimaging Initiative approach, emphasizing multimodal analysis.

## 1 BACKGROUND

Global population aging is driving a surge in dementia, now a leading cause of disability in older adults and a major societal burden.^1^ More than 50 million people currently have dementia, with 10 million new cases diagnosed yearly,^2^ predominantly in low‐ and middle‐income countries (LMICs).^2^ World Health Organization (WHO) projections indicate the prevalence could triple to 150 million by 2050, especially impacting rapidly aging societies like those in Africa.^2^ Within the next two decades, cases in the African region are projected to surge by up to 357%.^1^

Alzheimer's disease (AD) and mixed dementias are prevalent among individuals of African ancestry^3^ who also face a heightened prevalence of cerebrovascular disease.^3^ Given that brain pathology manifests early, beginning ≈ 15 to 25 years before dementia onset,^4^ implementing preventive measures in African populations is imperative.

Encouraging reports from the Lancet Commission on Dementia Prevention, Intervention, and Care estimates that as much as 40% of AD and related dementias (ADRD) cases could be prevented,^5^ primarily by addressing modifiable risk factors such as physical activity, cognitive engagement, smoking, diet, and vascular/metabolic health.^5^, ^6^, ^7^, ^8^ Comprehensive risk reduction strategies simultaneously targeting multiple risk factors have been recommended by the WHO as optimal approaches,^7^ recognizing the limitations of single‐domain interventions.^7^ Outside of Africa, successful implementation of such multimodal approaches has been demonstrated,^9^, ^10^, ^11^ including in remote‐based intervention trials.^12^

Pioneering these studies is the landmark Finnish Geriatric Intervention Study to Prevent Cognitive Impairment and Disability (FINGER, ClinicalTrials.gov: NCT01041989) multidomain lifestyle modification trial, which demonstrated significant improvements in cognitive, brain, and physical health outcomes 2 years after the intervention.^13^ The FINGER intervention also enhanced quality of life, reduced disability development, and was deemed cost effective.^14^, ^15^, ^16^ Researchers globally are applying and advancing this collaborative, novel approach within the Worldwide‐FINGERS (WW‐FINGERS) network,^17^ with promising reports on intervention effectiveness emerging.^14^, ^15^, ^16^, ^18^ However, to ensure the effectiveness of dementia prevention strategies, particularly in LMIC contexts, it is essential to consider contextual factors such as socio‐cultural beliefs and perceptions, knowledge, and economic influences.

Multiple studies from Africa report high prevalence of modifiable factors for ADRD including physical inactivity, low formal education, inadequate social interaction, unhealthy diet, tobacco use, and increasing alcohol consumption contributing to higher rates of non‐communicable diseases (NCDs) related to vascular and metabolic disorders (see Akineymi et al.,^3^ Bassil et al.,^19^ Ogunniyi et al.,^20^ Gureje et al.^21^, and Appendix SB). Despite these epidemiological insights, interventions evaluating the effectiveness of culturally sensitive and scalable approaches for reducing dementia risk in Africa are unavailable. While dementia risk factors have been identified in high‐income countries (HIC), there has been insufficient evaluation of their heterogeneity in LMICs and the disparities that may influence these factors. Additionally, integrating these efforts within community‐based health system enhancement programs is lacking. Another related issue is the necessity to build the capacity of researchers in LMICs to address these emerging challenges and play a key role in leading such initiatives.

**FIGURE 1:**
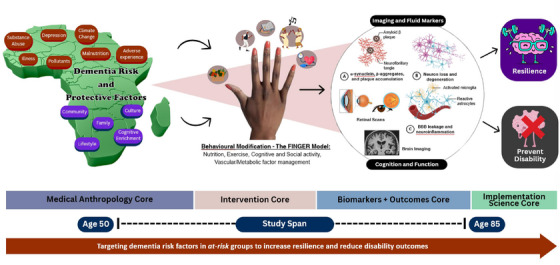
Conceptual diagram of the Africa‐FINGERS Project. Multideterminant framework for dementia risk reduction in Africa via pioneering precision prevention approach.

To address these gaps, we have developed the Africa‐FINGERS program,^22^ a multi‐country initiative to promote brain health in Africa (Figure 1). Africa‐FINGERS marks the inaugural effort to coordinate and implement culturally informed multidomain interventions to reduce AD/ADRD risk across the continent, with a strong emphasis on sustainability of these interventional and novel strategies for assessment, management, and prevention. The regional brain health promotion initiative, part of the WW‐FINGERS network, collaborates with local stakeholders to conduct culturally informed risk reduction trials and health systems programs to prevent cognitive impairment and promote healthy aging in Africa.

The project will address modifiable risk factors described by the Lancet Commission and WHO policies on addressing NCDs, with active involvement from indigenous community members, and guided by expert frameworks^7^, ^23^ to:

1. Co‐design a contextual multimodal lifestyle modification protocol with local stakeholders and collect data on intervention efficacy, biomechanisms, and cost effectiveness; and
2. Promote sustained intervention scalability through community‐wide implementation, while harmonizing methods for adoption across partner countries and similar demographics.

Here, we describe the innovative strategy used in the Africa‐FINGERS project, and its integration within the WW‐FINGERS framework.^23^ We further discuss how the multimodal analyses detailed within the research protocol align with the core principles of landmark global initiatives such as the Alzheimer's Disease Neuroimaging Initiative (ADNI) program.^24^

## 2 METHODOLOGICAL APPROACH

The Africa‐FINGERS project is a 5‐year initiative, set across vanguard sites in Kenya and Nigeria. It will include the first dementia risk reduction implementation randomized control trial (RCT) in these regions, adopting a mixed methods analytical approach and incorporating the RE‐AIM (Reach, Effectiveness, Adoption, Implementation, and Maintenance) framework for equitable implementation.^25^ The RCT will leverage established cohorts for efficient recruitment, and is registered with Pan African Clinical Trial Registry under unique identification number for the registry (PACTR202407499314703). Africa‐FINGERS is culturally informed, and the trial protocol will be co‐designed with local stakeholders, aiming for translatable interventions, and serving as a model for prevention research in LMIC settings.

### 2.1 RCT protocol development via co‐design methods

Using a range of participatory methodologies, beginning with a community‐based participatory research (CBPR) approach, the team is developing a multimodal intervention protocol for dementia prevention in African populations that uses participatory methods, co‐design workshops, and collaborative stances combining diverse disciplines, expertise, research–policy–practice partnerships. Integrating CBPR principles, the involvement of local stakeholders from the outset ensures that community values and priorities shape the intervention and promote sustainability. We further leverage insights from WW‐FINGERS studies. A diverse team from the Africa‐FINGERS Executive Steering Committee, proficient in national languages (Swahili, Yoruba, Igbo, Hausa) and cultures, will oversee the design process. This team includes WW‐FINGERS Senior investigators, alongside local and international domain experts. Following necessary ethical approvals from each research site, six focus group discussions in Kenya and Nigeria will explore culturally suitable lifestyle interventions, adapting elements from prior studies and identifying locally significant risk factors. Prior to engaging the community stakeholders in the co‐design workshops, we will obtain national and local ethical approvals for all arms of the project, including the co‐design phase, trial, and evaluation of intervention sustainability. Only individuals who provide informed consent will be invited to participate.

Building on close collaborations with community advisory boards (CABs), the project will consider additional risk factors such as smoking cessation, access to health aids, and family support structures, that are pertinent to the local context. These factors will inform customization of the Africa‐FINGERS protocol to align with global dementia prevention research, while being locally relevant. Our ongoing efforts uniquely leverage expertise of anthropologists, sociologists, cultural psychologists and leaders within the two countries to develop these participatory research processes. In‐depth interviews (IDIs) with expert stakeholders in each country will further refine the cultural suitability of intervention strategies. Feedback from these interviews will help tailor monitoring forms to local contexts and identify key intervention themes. Through convening structured workshops across urban and rural sites in both countries, the team will engage stakeholders such as village chiefs, health‐care providers, and policymakers in a sequence designed to minimize power differentials and foster robust discussion. These workshops focus on contextualizing the intervention modalities, such as integrating traditional dance routines and nutritional guidance, and assessing trial feasibility, including local adaptations of outcome measures like the Intervention for Dementia in Elderly Africans Instrumental Activities of Daily Living (IDEA‐IADL)^26^ and the African Neuropsychological Test Battery (A‐NTB).^27^ The eventual components of the multidomain intervention (physical, cognitive, and health) are subject to community review and ongoing focus groups. Therefore, the perceived vagueness or diversity in these descriptions reflects this stage of the study pending outcomes of stakeholder engagement activities. Evidence from a RCT of a multidomain intervention in at‐risk older adults, affiliated with the FINGERS network, suggests that 15 to 20 multidomain sessions are optimally sufficient for cognitive benefit.^28^ Nevertheless, recommendations from community review and focus groups will better inform culturally appropriate interventions, frequency, and dosage. Through rapid thematic analysis of workshop outcomes, the team purports to create a theory of change framework^28^ that will guide and evolve the questions around barriers and intervention development. The final workshops will harmonize perspectives and prioritize interventions, ensuring the development of a culturally informed, community‐upheld intervention protocol. This approach ensures compliance, and respects and upholds community values throughout the research process.

#### 2.1.1 Overall inclusion/engagement and schedule of events

Potential participants identified from existing cohorts as “at risk” of dementia will undergo a screening visit to assess eligibility based on inclusion criteria including age (50–85 years), Cardiovascular Risk Factors, Aging, and Dementia (CAIDE) Dementia Risk Score ≥ 6 points, cognitive performance, language proficiency, capacity to consent, and stable medication use. Exclusion criteria will include for example, dementia, impaired decision making, neurological diseases, and health conditions hindering safe trial participation. Eligible participants will proceed to a baseline visit, at which randomization to a structured or self‐guided (control) multimodal intervention occurs. Assessments will occur at 6, 12, and 24 months, covering primary/secondary outcomes, cardiovascular/metabolic risk factors, and safety parameters. The RCT lasts 24 months. Illiterate participants will not be excluded. Illiteracy rates are high in the study settings and are commonly associated with risk factors for age‐related cognitive decline, such as multi‐dimensional poverty.^29^ Therefore, it is essential to use tools and materials with very low to no literacy demands. Consent procedures will allow for oral explanations and thumbprints in lieu of written signatures. Indeed, study assessments such as the neuropsychological tests were selected based on their low literacy demands and are currently being pilot tested across several funded studies in Kenya and Nigeria to model literacy effects. This will inform refinement strategies by selecting tools with the lowest literacy effects. Similarly, intervention materials will be designed to cater to illiteracy by including illustrations and explanatory visual schemas. This approach ensures that the study selection phase does not unfairly exclude individuals who may be at greatest risk for dementia.[Fig alz14344-fig-0002]

#### 2.1.2 Enrollment strategy in participating sites

Participants (300 from Kenya and 300 from Nigeria) will be recruited from pre‐phenotyped observational cohorts, enabling swift enrollment of at‐risk individuals based on dementia risk factors, including family history, and limited social engagement. Specifically in Nigeria, the PERODEM and ADIPRES‐N studies led by Africa‐FINGERS co‐Is (DO and VU), which have detailed dementia risk data, will be leveraged. This prior work facilitates streamlined recruitment through pre‐existing databases and established relationships, ensuring a diverse participant pool and simplified informed consent. In Nairobi, Kenya, four AD‐related cohorts that are led by the Africa‐FINGERS Chief Investigator will be targeted, including FemBER Africa, Brain Resilience Kenya, AD‐Detect Kenya, and the Brain health Assessment Protocol Kenya project, totaling up to 1200 potential participants. Further, established collaborations with local organizations such as Nivishe Foundation, Futboll Mass, and the Ismail community, will support rapid recruitment for the co‐design workshops. At the Kilifi site in Kenya, we will augment potential recruits from the LOSHAK study, led by Africa‐FINGERS co‐I (AN), with participants from the KRHDSS cohort, a comprehensive demographic and health registry managed by Aga Khan University and local health teams. Recruitment will be door to door, using the established network of community health unit (CHU) leaders and volunteers. Additional recruitment methods will involve distributing flyers in clinics, religious venues, and community centers, as well as collaborating with non‐governmental organizations (NGOs) serving older populations. Purposive sampling will ensure balanced representation across various demographic factors such as sex, ethnicity, age, and socio‐economic status. Enrichment strategies will target socio‐economically deprived areas and specific community groups, collaborating with governmental and NGOs to ensure a diverse and representative sample reflective of Kenya and Nigeria's multi‐ethnic population. After enrollment and consent, the 600 participants will be randomized 1:1 to either the self‐guided (control) or the structured (active) multimodal group receiving the FINGER‐based intervention.

#### 2.1.3 Intervention delivery

**FIGURE 2 alz14344-fig-0002:**
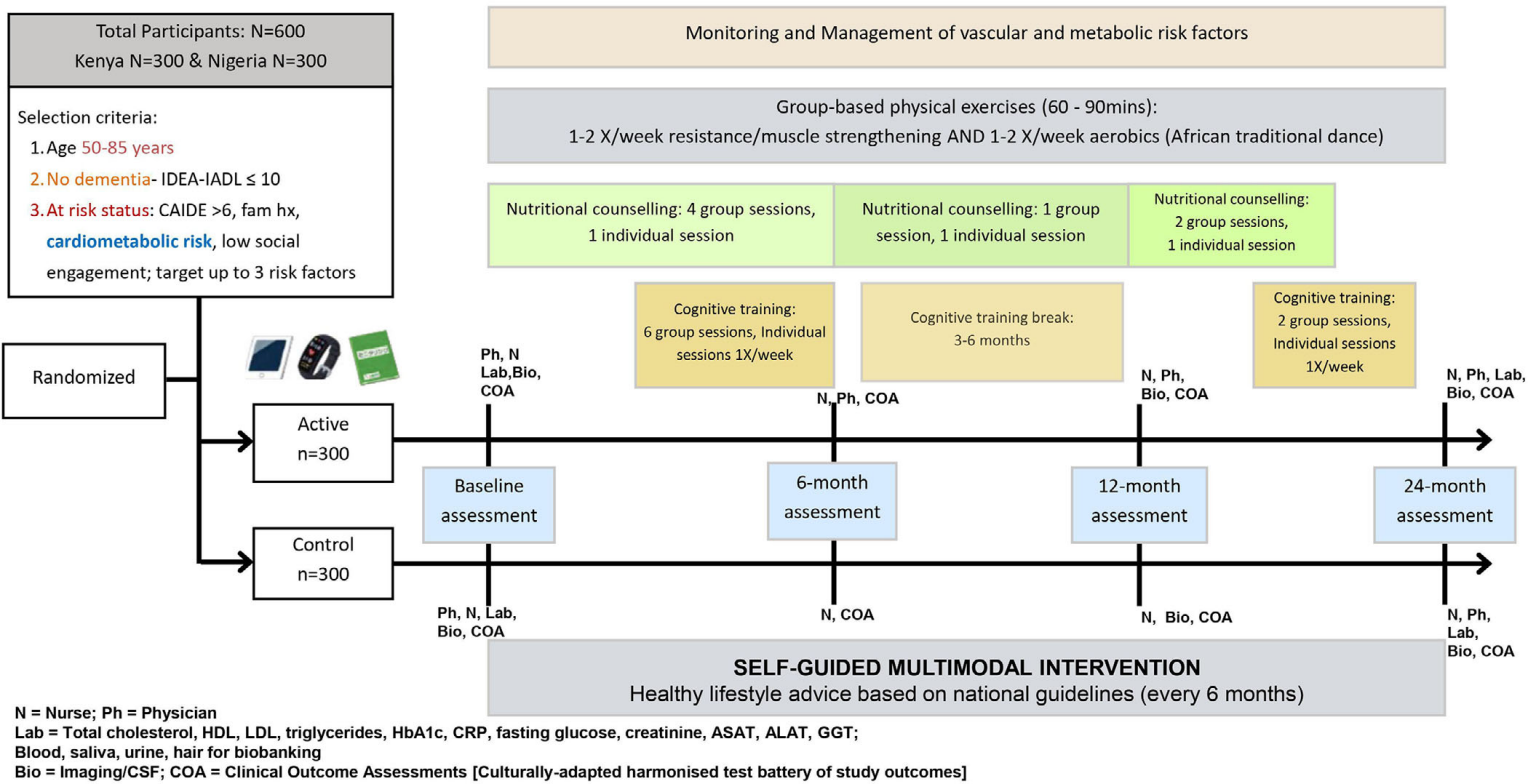
Proposed plan of the Africa‐FINGERS randomized control trial.

The structured multimodal lifestyle intervention includes distinct components: diet, physical activity, cognitive enrichment, and vascular/metabolic risk management, all integrated within a social engagement framework^30^ and co‐designed with community stakeholders (see Figure 2 for RCT schedule).

Aligning with WHO nutrition guidelines, the dietary intervention includes individual and group counseling sessions led by a trained nutritionist that will provide training on accessible and cost‐effective local food products, to promote healthy dietary habits. In LMICs, globalization has introduced high‐sugar, high‐salt, and processed foods, leading to obesity and type 2 diabetes. Rural communities often rely on starchy staple foods lacking in essential nutrients like omega‐3, vitamin B12, and vitamin C & E, crucial for brain function. Thus, personalized dietary advice will recommend fruit, vegetables, and protein sources like fish, chicken, dairy, and vitamin‐fortified cereals, while reducing sugar, salt, saturated fats, and alcohol. We will also adapt the African‐based Positive Deviance/Hearth approach^31^ for elderly nutrition education and food preparation. The physical activity intervention incorporates traditional dance routines, culturally significant in Africa, supervised by a physiotherapist or professional trainer, occurring at least once a week. For participants with mobility issues, alternative exercises will be recommended. Small group sessions promote social engagement and physical activity that will be objectively assessed using pedometers or actigraphy. For cognitive enrichment, accessible methods such as learning a new language, arts/skill‐based interventions, traditional games, and personalized goal‐setting and problem‐solving tasks based around financial literacy and well being will be used.^32^ These methods are culturally adaptable and aim to enhance neuroplasticity. Cardiovascular and metabolic risk monitoring and management involves five individual appointments with the study physician or nurse, including assessments, feedback, medical advice, and motivational discussions. Focus areas include smoking cessation and reduced alcohol consumption, stress, diabetes, and hypertension management. Study physicians will monitor fasting blood glucose, HbA1c levels, and blood pressure, using new technologies like mobile‐delivered ecological interventions to support smoking/alcohol cessation. Appropriate medications will be prescribed as needed, via, for example, centralized and digitized pharmacy solutions. Social engagement will be facilitated through group meetings within the intervention programs.

Participants in the self‐guided multimodal intervention (comparator/control) group will receive health advice during assessment visits (baseline, 6, 12, and 24 months) to encourage independent lifestyle changes. Given the extremely limited access to health care for instance in rural areas, a wait‐list approach may be used regarding potential medications, if needed as part of the cardiovascular monitoring and management intervention.

Outcome assessors will be blinded to group allocation, and participants will be instructed not to discuss intervention activities during outcome assessment visits. Intervention compliance/adherence will be measured via attendance records at group intervention meetings (e.g., for physical, social, and cognitive activities), questionnaires on dietary changes/intake, subjective reports of satisfaction with progress to monitor skills/brain training, and regular vascular and metabolic risk monitoring (blood pressure, body mass index, and blood work). This strategy will ensure that we accurately interpret the trial findings by collecting robust data on intervention fidelity from both the investigators’ and participants’ perspectives.

To support a sustainable research infrastructure, we have included regionally validated scales to evaluate dementia risk factors and established biomarkers (e.g., neuroimaging, cerebrospinal fluid [CSF]), along with cost‐effective non‐invasive innovations (e.g., retinal imaging, blood, tear samples, saliva, digitized cognitive tests, wearable devices) that show great promise as tools for earlier ADRD detection. The following techniques, assessments, and methods will be used, albeit subject to co‐design.

For biomarkers, we will collect the following: (1) Genetic analyses will involve whole genome sequencing of genomic DNA extracted from whole blood. Polygenic risk score (PRS) analysis will be conducted using multi‐ancestry approaches. Further investigations will focus on ADRD‐related genes, including amyloid precursor protein/amyloid beta cleavage pathways, with emphasis on African‐specific risk variants (e.g., apolipoprotein E [*APOE*] {E3:R145C}; *TREM2*; *ABCA7*, *BDNF* {Val66Met}). (2) In a subset of participants (*n* = 150) age/sex‐matched, structural and functional brain magnetic resonance imaging (MRI) as well as fluorodeoxyglucose (FDG) positron emission tomography (PET) will be performed using scan protocols optimized from ADNI^24^ (Table 1/Appendix SC). CSF AD biomarkers are also planned for consenting participants. (3) Retinal imaging^33^ will involve modified scanning laser ophthalmoscope and ultrawide‐field laser scanning ophthalmoscope to detect retinal amyloid deposition and monitor ophthalmological changes. (4) Urine, blood (whole: serum, plasma, peripheral blood mononuclear cells and RNA; finger prick‐derived), tears using Schirmer strips,^33^ hair, and saliva samples will be collected and processed for genotyping, clinical lab assessments, and AD‐related biomarkers, among others. (5) Physiology assessments involve digital phenotyping using wearable consumer sensors (FitBit Inspire 3) to monitor physical activity, sleep, and autonomic function and stress over a 14‐day period.

**TABLE 1:** Africa dementia imaging protocol modalities and alignment with Alzheimer's Disease Neuroimaging Initiative.

Sequence	Acquisition mode	Resolution (mm)	TR/TE/TI (ms)	TA (min)	ADNI aligned
**Core**					
T1w	3D—sagittal	1 × 1 × 1	7.3/3.3/935	6:50	Same
T2w	2D—sagittal	0.94 × 0.94 × 3	3000/100	5:24	Same
T2‐FLAIR	3D—sagittal	1 × 1 × 1.2	4800/275/1650	5:07	Slightly increased spatial resolution to 1 mm isotropic
T2 Star	2D—axial	0.8 × 0.8 × 4	650/20	4:29	Same
DWI^*^ (30 directions)	2D—axial	2 × 2 × 2	9916/86	6:53	Increase in the number of directions to 64 on 3T and same on 1.5T.
rsfMRI (BOLD)	2D—axial	3.5 × 3.5 × 3.5	3000/30	10:00	Same
PASL^**^	2D—axial	3 × 3 × 4	5000/16 (PLD = 2000)	5:10	Similar with addition of 2 M0 calibration images in opposing phase‐encoding directions
**Advanced**					
CVR‐rsfMRI (BOLD)	2D—axial	3.5 × 3.5 × 3.5	3000/30	5:00	New and introduced here for low‐cost assessment of cerebrovascular risks

*Note*: Summary of ADIP MRI modalities and alignment with ADNI. Parameters shown based on ADNI3‐Philips R3 protocol with an eight‐channel phased‐array head coil (https://adni.loni.usc.edu/methods/documents/mri‐protocols/).

Abbreviations: 2D, two‐dimensional; 3D, three‐dimensional; ADIP, Africa Dementia Imaging Protocol; ADNI, Alzheimer's Disease Neuroimaging Initiative; BOLD, blood‐oxygenation level dependent (300 volumes) using EPI imaging acquired with fixated eyes‐open conditions; CVR, cerebrovascular reactivity using breath‐hold maneuvers; DWI, diffusion weighted imaging using single‐shot echo‐planar (EPI) sequence; FLAIR, fluid‐attenuated inversion recovery; MRI, magnetic resonance imaging; PASL, pulsed arterial spin labeling with 30 labels and controls; PLD, post‐labeling delay; rsfMRI, resting state functional magnetic resonance imaging; TA, time of acquisition; TE, echo time; TI, inversion time; TR, repetition time.

^*^ *b*‐value = 0 and 1000 with two spin‐echo images acquired in opposing phase‐encoding directions (*b*‐value = 0 s/mm^2^ & 6 directions).

^**^M0 = proton density calibration scan acquired using ASL sequence with longer TR (> 7 seconds) and no label or background suppression. Two M0 images will be acquired in opposing phase‐encoding directions with five repetitions/averages. All sequences except for are accelerated at a factor of 2.

Cognitive and functional outcomes are assessed using tools adapted for the region.^19^, ^27^, ^34^ These tests were chosen to align with other WW‐FINGERS sites, following consensus discussions among African neuropsychologists and the WW‐FINGERS Global Scientific Coordinating Center. All participants are screened for cognitive impairment using the locally validated IDEA cognitive screen and IADL, which together have an area under the receiver operating characteristic curve of 0.94 for Diagnostic and Statistical Manual of Mental Disorders Fourth edition dementia diagnosis.^34^ The full A‐NTB includes measures from the domains of memory, language, attention/speed, sensorimotor, and executive functions. Consistent with the WW‐FINGERS approach, a global composite score will be generated from all neuropsychological measures as the primary outcome variable, with rigorous examination of specific test psychometrics prior to inclusion. Additionally, domain‐specific composite scores will be generated as secondary outcome measures, and an actuarial approach adopted, using local normative data, to classify mild cognitive impairment (MCI), following the Jak/Bondi criteria.^35^ This flexible, data‐driven approach to generating primary and secondary neuropsychological outcome measures will result in locally valid and globally harmonized outcome measures.

Social determinants of health, such as socio‐demographics (age, biological sex at birth, identified gender, socio‐economic class [childhood and current], income, quantity, quality, and nature of education, occupational attainment/complexity, neighborhood characteristics [rural, urban, semi‐rural areas], social network and support, lifetime stress and resilience), will be assessed via culturally adapted measures recently demonstrating construct validity in African samples (e.g., Connor‐Davidson Resilience Scale and Life Events Scale). Adapted items from the Social and Structural Life‐Courses Influencing Aging and Dementia (SS‐DIAD) battery will characterize social and environmental conditions across the life course relevant for health and quality of life. Questionnaires will be designed in close collaboration with community stakeholders and regional experts to capture the ethnic and linguistic diversity of the participating countries (e.g., in Nigeria: Igbo, Yoruba, Hausa, Ijaw, Edo, Efik, Tiv, Asian Nigerians, etc.; and Kenya: Kikuyu, Luhya, Luo, Kalenjin, Kamba, Kisii, Maasai, and Meru peoples as well as other minorities, such as those of Asian and European ancestry).

Clinical/health/lifestyle‐related factors will be captured via structured interviews to probe medical history and medication ‐use, history of traumatic brain injury, current smoking status and history, diet, physical activity, social engagement, and sleep, using culture‐specific questionnaires, co‐developed with the stakeholder team.

Previous work in Africa has revealed significantly lower risk for incident dementia in men versus women.^20^ For the sub‐study investigating biological sex and gender influences on dementia prevention mechanisms, data on sex‐specific reproductive health factors will be collected. These include history and age at time of reproductive surgical procedures (e.g., males: vasectomy; females: hysterectomy, ovariectomy) and hormone use (such as fertility treatments, acne medication, birth control, hormone replacement therapy [HRT] use: current use, past use, age at first use, duration in months, HRT type); puberty onset, number of children, fertility issues, and history of sexually transmitted diseases. For female participants, data on current menopausal status if known (pre‐, peri‐, post‐menopause) and symptoms, menopause transition history (if applicable); age of menarche onset and termination (if applicable), recent menstrual cycle history (if applicable), number of pregnancies and births (including fields for abortions, miscarriages, stillbirths), and age of pregnancies and births are collected.

The Community Health Promoters and Community leaders have already established trust within the community, and will assist in maintaining contact and motivating participants throughout the study. We offer flexible appointment times and accessible locations to accommodate participants’ schedules, provide transportation assistance, and reimburse travel expenses to reduce barriers to participation. Additionally, participants receive regular updates about their health progress and any preliminary findings of interest. Collaborating with established CABs, who reside in the communities where our studies will be conducted, will further ensure sustained relationships and participation. All enrolled participants are invited to join the Africa‐FINGERS brain health register, which serves as a preparedness cohort for individuals consenting to future brain aging–related studies. Continuous engagement with members of the Africa‐FINGERS register is ensured through retention activities, including bi‐annual newsletters and annual participant engagement seminars. Additionally, study teams organize virtual educational seminar series on brain health. Long‐term goals include establishing new diasporic cohorts in the UK/EU and United States, complementing our Africa‐based cohorts, and enhancing Western ones. Through these initiatives, our participants will join a global brain health movement, collaborating on research and engaging in public outreach.

### 2.2 Data management and statistical analysis

The Africa‐FINGERS RCT pioneers exploring dementia prevention mechanisms in African populations. It aims to link intervention benefits on biomarkers with cognitive and functional improvements, thereby offering a comprehensive approach to dementia prevention research in Africa. All data management will be performed in accordance with the International Conference for Harmonisation of Technical Requirements for Pharmaceuticals for Human Use guidelines. Data files and biological samples will be stored and managed in compliance with relevant national regulations and laws regarding data protection and privacy. Specifically, all data will be stored using the Research Electronic Data Capture (REDCap) platform, a secure system developed by Vanderbilt University and the National Institutes of Health. The REDCap platform ensures data security and is maintained on the university IT server, which is equipped with robust security measures to safeguard data integrity and confidentiality.

Power calculations for Africa‐FINGERS are based on findings from the FINGER study and other WW‐FINGERS trials. The minimum sample size required was determined using the sample size formula for comparing two means, focusing on the mean change in cognition for persons with *APOE* ε4 carrier status from the FINGER trials (control mean change 0.01 [standard deviation (SD) = 0.38] vs. intervention mean change 0.19 [SD = 0.34]). The calculated minimum sample size is 253 participants per group, resulting in a total of 506 participants. With an estimated sample size of *N* = 600, we anticipate adequate power for our study, as the minimum of 506 participants ensures 80% power at a 5% significance level.

This sample size accounts for the heightened dementia risk observed in individuals of African ancestry compared to Caucasians, as well as the expected increased adoption and adherence to the ethnocultural intervention, as demonstrated in the SINGER trial.^36^

A summary of the Africa‐FINGERS primary, secondary, and exploratory outcomes is provided in Table 2. The primary neurocognitive outcome measures the composite score of the extended A‐NTB, adapted from the FINGER trial, focusing on memory, executive function, and processing speed. Tests are tailored for African populations and translated into local languages. The effectiveness of the FINGER‐based multimodal lifestyle intervention versus a self‐guided intervention will be evaluated over 24 months. Secondary outcomes include changes in cognitive domains, functioning levels, healthy lifestyle changes, cardiovascular and metabolic risk factors, diet, physical activity, physical functioning, and other measures such as depressive symptoms and quality of life. Exploratory outcomes investigate dementia/AD biomarkers using CSF, blood, saliva, retinal imaging, brain MRI outcomes, and FDG‐PET assessments. Subgroup analyses will examine geographical and sex/gender differences, particularly higher dementia risk in women.

**TABLE 2:** Experimental outcomes of the Africa‐FINGERS randomized control trial.

Primary outcome: The primary neurocognitive outcome focuses on the composite score of the extended African Neuropsychology Battery (A‐NTB), adapted from the FINGER trial. Memory, executive function, and processing speed domains will have separate composite scores derived from individual tests harmonized with other FINGER projects and cohorts like ADNI. These tests, validated or under validation in African populations, accommodate low education and literacy levels, and are translated into local languages. The composite A‐NTB score will standardize 12 test components to their baseline mean and standard deviation to obtain *Z*‐scores, averaging these to form domain‐specific and overall scores. Included tests are the Word List‐Learning Test, Brave Man Story Recall, Relational/Associative Memory, Animal Category Fluency, Multilingual Naming Test, Nigerian Matchstick Task, Four Mountains Test, Tab CAT Match Test, Tab CAT Flanker Test, Tab CAT Set‐Shifting, and Digit Span Test. The effectiveness of the FINGER‐based multimodal lifestyle intervention compared to a self‐guided intervention will be evaluated over 24 months.
Secondary outcomes: (i) Multi‐domain Cognition: Changes in individual cognitive domains using the A‐NTB. Composite scores will be calculated if at least three of five subtest summary scores for executive function, two of three for processing speed, and three of six for memory are available. (ii) Functioning Level: Assessed with the Intervention for Dementia in Elderly Africans Instrumental Activities of Daily Living scales and the Clinical Dementia Rating Sum of Boxes tool. (iii) Healthy Lifestyle Changes: Assessed via a Healthy Lifestyle Index covering physical activity, diet, cardiovascular risk factors, and social and cognitive activity. (iv) Cardiovascular and Metabolic Risk Factors: Monitored changes in BMI, waist/hip ratios, blood pressure, blood lipids, and glucose levels. (v) Diet: Evaluated through a composite adherence score based on the FINGER trial, focusing on local and cost‐effective foods. (vi) Physical Activity: Assessed through self‐reports, accelerometers, and anthropometric measurements. (vii) Physical Functioning: Measured with culturally appropriate tests. (viii) Other Outcomes include depressive and anxiety symptoms, stress‐related symptoms, sleep problems, quality of life, resilience, and flourishing, along with health resource use.
Exploratory outcomes: Investigation of dementia/AD biomarkers such as CSF and blood biomarkers (Aβ42/40 ratio, phosphorylated tau, brain‐derived tau, neurofilament light chain, and glial fibrillary acidic protein) is planned. Brain MRI outcomes (volumetry, cortical thickness, white matter lesions) and FDG PET will assess cerebral glucose metabolism. Further, amyloid and tau accumulation via CSF biomarkers and AD‐related blood markers are explored. Although the *APOE* ε4 variant does not increase AD risk in Africans, molecular signatures predicting cognitive/functional response to interventions will be studied. Integrating GWAS data with pQTLs will identify genetic variants associated with circulatory AD biomarkers, characterizing pQTL variants related to ADRD. Mendelian randomization will determine causality, thus identifying targets for disease prevention and treatment.

*Note*: Summary of primary, secondary, and exploratory outcomes in the Africa‐FINGERS RCT.

Abbreviations: Aβ, amyloid beta; AD, Alzheimer's disease; ADNI, Alzheimer's Disease Neuroimaging Initiative; ADRD, Alzheimer's disease and related dementias; *APOE*, apolipoprotein E; BMI, body mass index; CSF, cerebrospinal fluid; FDG PET, fluorodeoxyglucose positron emission tomography; GWAS, genome‐wide association study; MRI, magnetic resonance imaging; pQTL, protein quantitative trait loci; RCT, randomized controlled trial.

Mixed‐effects regression models with maximum likelihood estimation will analyze cognition change based on randomization group, time, and group × time interaction, adjusting for the trial site. Models with a quadratic term will also be explored. Primary efficacy analysis will focus on the modified intention‐to‐treat (mITT) population (all randomized participants with at least one follow‐up). Secondary and sensitivity analyses will involve the intention‐to‐treat (ITT) population (all randomized participants) with multiple imputations. For binary outcomes, either a discrete‐time hazard regression model or a logistic regression model will be applied. Linear regression will assess continuous outcomes with baseline and 24‐month visit measurements. Subgroup analyses by factors like age, sex, baseline cognitive and functional level, and intervention adherence will be conducted. Dementia as an outcome is not feasible after 24 months as evidenced from FINGER;^13^ however, extended follow‐ups will be conducted through national registers and brain banking consents. Qualitative research, such as post‐intervention IDIs, will explore participant experiences, aiding in refining the intervention strategy.

The RCT will also evaluate the feasibility and acceptability of the culturally validated FINGER intervention in sub‐Saharan Africa (SSA), including adherence metrics. Feasibility of innovative technologies will be assessed, addressing in‐country capacity development goals, including digitized cognitive assessments, non‐invasive biosample collection, high‐throughput biomarker evaluations, and advanced biorepository development.

### 2.3 Cost‐effectiveness evaluation

A two‐stage Bayesian framework will assess the cost effectiveness of the Africa‐FINGERS intervention. The first stage develops a simulation model based on country‐specific data to estimate cost effectiveness relative to local “willingness‐to‐pay” thresholds.^37^ An extended cost‐effectiveness analysis considers health financing and equity.^38^ The second stage designs a within‐trial cost‐effectiveness analysis, collecting real‐world data on quality‐adjusted life years, resource use, and costing methodologies. This comprehensive approach informs decision making and budgeting for dementia prevention services.

### 2.4 Health and community systems implementation

**FIGURE 3:**
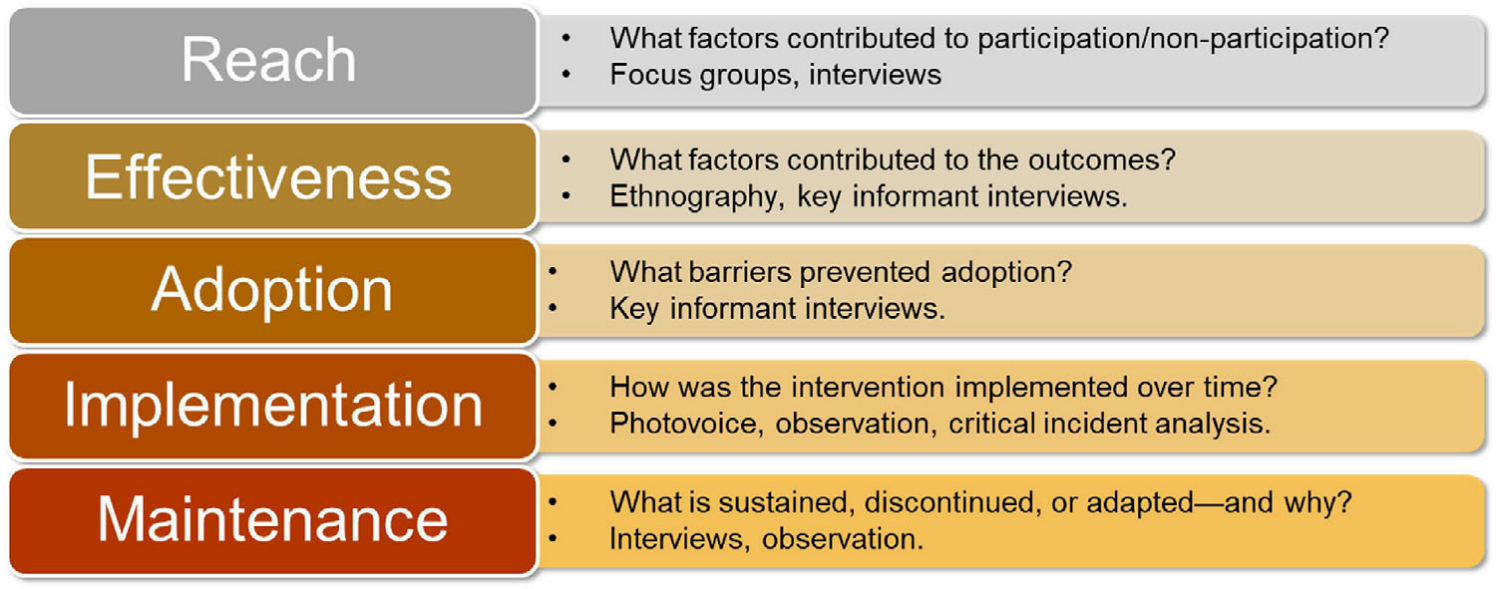
Africa‐FINGERS implementation plan adopting RE‐AIM framework. RE‐AIM, Reach, Effectiveness, Adoption, Implementation, and Maintenance.

Africa‐FINGERS will integrate a health promotion strategy into local and national networks, disseminating project lessons widely and continuously throughout the study period and beyond (Figure 1). This involves various academic and public outreach methods like peer‐reviewed articles, workshops, seminars, radio shows, and digital media (e.g., dedicated webpages, social media platforms, webinars, and online forums). Collaboration with regional partners will sustain momentum and empower health‐care practitioners and patients to manage dementia risks. Brain health literacy campaigns and stakeholder engagement aim for community empowerment, measured by pre‐ and post‐campaign surveys (Figure 3).^25^ Continuous dissemination and support from regional^39^ and global partners ensure project sustainability.

## 3 DISCUSSION

**FIGURE 4:**
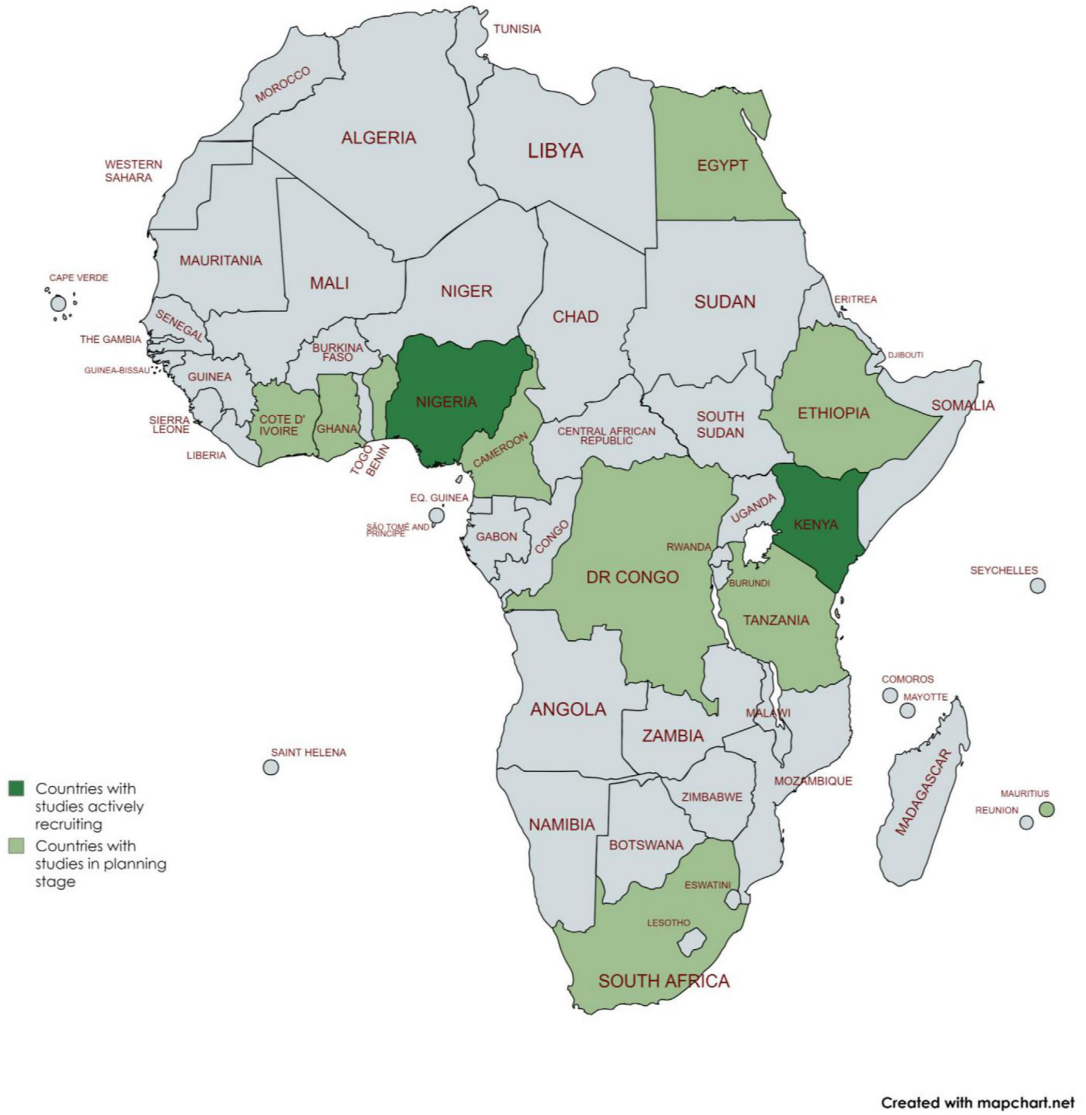
Participating countries in the Africa‐FINGERS program. African countries currently involved in the first Worldwide‐FINGERS program in Africa adopting CBPR approach toward culturally informed multimodal interventions with bespoke implementation and integration strategy. CBPR, community‐based participatory research.

The Africa‐FINGERS Project aims to develop and test strategies to reduce risk of ADRD in Africa using a culturally informed, multimodal precision prevention framework. As part of the WW‐FINGERS network,^23^ the project leverages established global partnerships and resources to build and sustain neuroepidemiology research and clinical trials in Kenya and Nigeria, with plans to extend to partner African countries (Figure 4), thereby fostering academic and research infrastructure development in the region.

### 3.1 Africa's first culturally tailored dementia risk reduction initiative

Adopting a CBPR framework, Africa‐FINGERS is the first culturally sensitive dementia risk reduction intervention developed within Africa, with a strategy informed by the landmark FINGER model. The multimodal lifestyle intervention will be tailored to fit the local cultural context and population. The project tests the hypothesis that effective and sustained prevention of cognitive impairment (CI) and dementia is most likely achieved via a multimodal precision prevention model within pre‐established risk groups. Furthermore, we anticipate that addressing the complexity and heterogeneity of ADRD, by targeting multiple risk factors affecting disease mechanisms and aging‐related processes, will expedite the development of suitable strategies for ADRD prevention among Africans.

While these objectives are distinctive within African dementia research, international studies have explored similar goals, and adapted FINGER interventions to local cultures successfully.^18^, ^36^, ^40^, ^41^ Comparisons between trials replicating the original FINGER model and those using adapted protocols have shown similar adherence rates. Notably, trials with culturally adapted protocols showed significant differences between the intervention and control groups, including in some cognitive domains similar to those studied in the FINGER trial.^18^, ^36^, ^40^ Africa‐FINGERS enhances existing models by integrating targeted management of both FINGER‐recommended and contextual risk factors, using a semi‐flexible intervention delivery approach that empowers participants to choose additional behaviors to modify. This model also involves decentralized trial methods, incorporating co‐design mechanisms from CABs and engaging the support and involvement of community health workers (CHWs) in the research process. Similar approaches have been undertaken in NCD management initiatives in the region. For example, the Scaling‐up Packages of Interventions for Cardiovascular Disease Prevention in Selected Sites in Europe and SSA: Implementation Research (SPICES Project) in rural and semi‐urban African settings reported the acceptability of lifestyle recommendations for NCD prevention when delivered via CHWs.^40^ Several considerations for the long‐term adoption of healthy behaviors were identified, which have been integrated into the Africa‐FINGERS program, including concerns regarding the implications of risk identification. Consequently, the project's control group may adopt a wait‐list design, allowing for potential extension of the protocol to control group participants should significant risk reduction be achieved in the intervention group. Additionally, the control arm will be examined to assess the impact of self‐guided health and risk factor management for dementia prevention. Future efforts will monitor and evaluate both groups, with particular focus on the sustainability of behavioral modifications by evaluating the continued adoption of health‐promoting behaviors.

### 3.2 Biophenotyping in African populations: Alignment of Africa‐FINGERS with global dementia research initiatives

Biological risk factors for identifying ADRD risk have traditionally been informed almost exclusively from HIC cohorts, necessitating validation in other populations.^3^ Indeed, biomarker‐access inequities pose a significant challenge in Africa, potentially contributing to under‐ or misdiagnosis of ADRD.^3^ Despite high dementia risk among African populations,^1^, ^3^ there are limited studies on AD‐related biomarkers in these groups. This gap persists despite advancements in imaging and biofluid‐based biomarkers that could revolutionize dementia risk assessment in LMICs. There is a clear need to bolster diagnostic capacity in Africa and discover novel multimodal preventive therapies that combine pharmacological and non‐pharmacological approaches. Africa‐FINGERS tackles these knowledge gaps by exploring biological causes and the impact of behavioral changes on cognitive health. The project will pioneer global efforts by linking lifestyle interventions to AD biomarkers in indigenous Africans.

Africa‐FINGERS will involve enhanced capture and analysis of biospecimens and neuroimaging modalities using advanced tools and techniques, which is poised to identify unique genetic and biological determinants of CI. The biospecimen capture protocol of Africa‐FINGERS uses comprehensive biophenotyping of study participants, carefully considering culture and context. To improve accessibility, we will also establish capillary finger‐prick sampling for dried plasma spots, enabling room‐temperature storage and broader application, particularly in remote areas. This will democratize access and examine the impact of FINGER interventions on ADRD biomarkers. However, given that many assessments may be unfamiliar, community sensitization before implementation will be essential. Therefore, the initial phase of the study will entail focus groups with CABs to gauge attitudes, perceptions, and the acceptability of planned sampling procedures (including brain donations for standardized neuropathologic assessments^42^), analytical processes, as well as material and data‐sharing plans—all aligned with the National Centralized Repository for Alzheimer's Disease and Related Dementias Alzheimer's Disease Center Fluid Biomarkers (ADCFB) and ADNI‐Neuropathology Core protocols.^43^, ^44^ These protocols, featuring best practice methods, are used by several AD research centers and global studies, including the longitudinal multisite ADNI program.^24^ Africa‐FINGERS similarly conducts enhanced biosampling,^24^ which will yield etiologic insights into disease biomechanisms to help unravel the complexity and heterogeneity of the AD syndrome.^45^

The Africa‐FINGERS project will develop a brain imaging protocol adapted from ADNI PET and MRI Core protocols (https://adni.loni.usc.edu/) that is suitable for the scarcely available technologies in Africa (i.e., 1.5T MRI scanners; Table 1). This will facilitate comparable dementia biomarker imaging in resource‐constrained settings. The Africa Dementia Imaging Protocol (ADIP; Appendix SC) provides comprehensive neuroimage acquisition and analysis procedures to characterize dementia risks in African populations, addressing these groups’ unique needs and vascular risk profiles. Research on retinal pathologies suggests that retinal imaging could be a non‐invasive, cost‐effective surrogate for AD‐related brain pathologies.^46^ Modern imaging techniques measuring retinal features (vascular parameters, layer thickness) and tear biofluid analysis show promise as ADRD biomarkers.^33^, ^46^ These methods could enhance specificity and selectivity through artificial intelligence–based analysis and complement global AD studies.^47^

In these ways, Africa‐FINGERS culturally adapts fluid and imaging biomarker harmonization strategies from global studies toward multimodal assessments of CI and ADRD risk in diverse populations.^23^, ^24^

Apart from inequitable access to fluid and imaging biomarkers, the availability of valid cognitive assessment tools that suit the region's vast socio‐cultural, educational, and linguistic diversity remains a significant challenge. Neuropsychological science and professional practice in Africa are still in their infancy, grappling with unique public health barriers such as limited health‐care resources, food insecurity, and prevalent traditional beliefs about disease.^48^ Although recent developments have been promising,^19^, ^26^, ^49^, ^50^, ^51^ the integration of these measures in multiple assessments over time, complemented with rich phenotypic data to substantiate disease diagnosis, remains scarce. Furthermore, existing measures often lack alternative versions for longitudinal follow‐up, which are necessary to avoid practice and familiarity effects. Similarly, evidence of their efficacy in detecting interventional effects is still elusive. The Africa‐FINGERS cognitive battery was strategically harmonized with established ADRD cohorts, such as the ADNI initiative, for neuropsychological domains of interest, with composite indices created that may be comparable across studies. Our methodology ensures that tests are culturally appropriate and/or adapted through extensive stakeholder review. This includes developing local test adaptations where necessary, training local researchers and professionals, and actively engaging with communities to ensure the relevance and acceptance of these tools. Our cognitive test protocol is designed to address issues related to practice effects by using tasks with alternative versions. We will rely on composite indices and endpoints, rather than specific measures, to ensure robustness to cross‐cultural variation and harmonization with WW‐FINGERS. By prioritizing these strategies, we foster a more equitable and effective implementation of neuropsychological assessments across diverse African contexts, ultimately enhancing diagnosis and treatment outcomes.

Similarly, the Africa‐FINGERS multi‐modal lifestyle intervention, an adaptation of the successful FINGER program, is tailored to align with local culture and context across the African settings. This customization will promote a culturally sensitive, practical, and sustainable intervention. Engaging local leaders and health‐care providers in tailoring the interventions, boosts legitimacy and acceptance of the program, ensuring it respects local knowledge and practices. This approach is poised to make Africa‐FINGERS both a health‐improvement initiative and culturally enriching experience.

### 3.3 Strengths and limitations

The Africa‐FINGERS program has several strengths, such as its cultural adaptation to local contexts, enhanced biophenotyping, a comprehensive approach targeting multiple risk factors, and strong community involvement, ultimately enhancing participant engagement and program acceptance. Additionally, it supports local capacity building—a key focus of the initiative—in a neglected area within dementia research in Africa and the cost‐effectiveness evaluations offer scalability of the program to different regions. To ensure effective capacity and infrastructure building and development, the Africa‐FINGERS Academy will be established, offering comprehensive training for early‐stage researchers and staff on clinical research design, implementation, and analysis, including data, sample collection, intervention delivery and monitoring, aligned with Good Clinical Practice, alongside effective use of sample and data analytical platforms. Furthermore, to foster long‐term collaboration and capacity building, international collaborators, including industry partners, alongside senior in‐country teams will support and lead training and capacity‐building activities, aiming to empower local researchers and promote their independence. This approach will build and sustain research capacity in the participating African countries, ensuring that local researchers are well equipped to conduct and lead future studies.

However, the program will likely face several challenges including its dependency on variable local resources, challenges in collecting consistent and reliable data, and variability in efficacy due to the diverse socio‐economic, linguistic, and cultural backgrounds of the populations under study. Further, sustainability could be an issue without long‐term funding and integration into national health systems. Addressing these challenges is crucial for enhancing the program's effectiveness and sustainability, ensuring it delivers lasting benefits across diverse African communities.

## 4 CONCLUSION

In summary, the proposed goals of Africa‐FINGERS hold significant promise for enhancing research development and welfare across Kenya, Nigeria, and affiliated nations.

Through targeted impact areas encompassing novel risk identification/reduction strategies, sustainable health initiatives, and inclusive education, the program will culturally adapt and expand upon broader global initiatives on dementia risk identification and prevention like ADNI and FINGER to suit regional contexts.

The program addresses cognitive and physical health concerns and has significant potential to alleviate the effects of other age‐related chronic conditions at both the community and societal levels, through the co‐designed comprehensive multimodal lifestyle‐based prevention protocol tailored for individuals at elevated risk of dementia in an African setting. Moreover, the inclusive engagement of stakeholders, with a deliberate focus on empowering female candidates in science, technology, engineering, and mathematics roles and offering health education on CI and dementia, is poised to catalyze future collaborations in research and public health among the UK/Europe, United States, and African nations, thereby delivering tangible benefits to policymakers both within and beyond the region.

## AUTHOR CONTRIBUTIONS

Conceptualization: CU and MK; data acquisition and curation: all authors; formal analysis: AS and JS; funding acquisition: Africa‐FINGERS Study Team; investigation: all authors; methodology: all authors; project management and operational oversight: WK and RM; scientific oversight: CU and MK; writing—original draft: CU; writing—review & editing: all authors. Equity commitment: A major focus of the initiative is capacity building and LMIC partner leadership. We have a team comprising both LMIC and HIC researchers, supporting and leading this research collaboratively, as evidenced by our mix of LMIC and HIC researchers in the site PI and core‐lead teams (see Appendix A). As a partnership, we will endeavor to uphold the Leroi et al.^52^ framework for equitable and collaborative research partnerships, which outlines good practice. The Chief Investigators and site leads of the Africa‐FINGERS study, as well as the study Work Package core‐leads, will specifically monitor and ensure equity in research opportunities and contributions between LMIC and HIC researchers within the teams. They will also oversee the budget and data ownership to maintain transparency and fairness in resource allocation and data management.

## CONFLICT OF INTEREST STATEMENT

H.Z. has served at scientific advisory boards and/or as a consultant for Abbvie, Acumen, Alector, Alzinova, ALZPath, Amylyx, Annexon, Apellis, Artery Therapeutics, AZTherapies, Cognito Therapeutics, CogRx, Denali, Eisai, LabCorp, Merry Life, Nervgen, Novo Nordisk, Optoceutics, Passage Bio, Pinteon Therapeutics, Prothena, Red Abbey Labs, reMYND, Roche, Samumed, Siemens Healthineers, Triplet Therapeutics, and Wave; has given lectures in symposia sponsored by Alzecure, Biogen, Cellectricon, Fujirebio, Lilly, Novo Nordisk, and Roche; and is a co‐founder of Brain Biomarker Solutions in Gothenburg AB (BBS), which is a part of the GU Ventures Incubator Program (outside submitted work). IL receives unrestricted research funding from OPTOS Plc. The other authors declare no competing interests. Author disclosures are available in the supporting information.

## CONSENT STATEMENT

The project protocol has been submitted to the Imperial College UK Research Ethics Committee (ICREC). Independent review by the institutional review boards at all participating local sites will be conducted and approvals obtained prior to project initiation. All study participants will provide informed consent to participate in the studies described.

## Supporting information



Supporting Information

Supporting Information

## Data Availability

After a brief embargo period, all relevant data from this study will be made available upon study completion through the Alzheimer's Disease Data Initiative platform. Researchers may request access through application to the Chief Investigators for review by the data management executive committee.

## APPENDIX A COLLABORATORS

### A.1

Africa‐FINGERS Investigative Team Members: study roles and affiliations at time of project initiation (in alphabetical order)

**Adedoyin Ogunyemi^1,2^**: Implementation Core co‐Lead, Lagos‐Nigeria intervention trial PI; **Rufus Akinyemi^2,3^**: Intervention Core co‐Lead**; Alina Solomon^4‐7^**: Data Analytics Core Lead**; Anthony Ngugi^8^**: Multi‐PI Kilifi, Kenya; **Celeste A. de‐Jager Loots^5,6^**: Intervention Core co‐Lead**; Chinedu T. Udeh‐Momoh^2‐5,9,10,11^**: Chief Investigator (CI) and co‐Chair of the Africa‐FINGERS Executive Steering Group; **Cyprian Mostert^9^**: Health Economics and Policy Implementation co‐Lead**; Dimitra Kafetsouli^3^**: Biomarkers Ophthalmology Core Co‐Lead**; Dominic Trepel^2,12^**: Health Economics and Policy Implementation co‐Lead**; Edna Bosire^9^**: Implementation Core co‐Lead**; Gloria Chemutai^9^**: Communications Core co‐Lead (Kenya)**; Imre Lengyel^13^**: Biomarkers Ophthalmology Core Co‐Lead; **Jasmit Shah^9^**: Biostatistician, Data Analytics Core‐co‐Lead**; Karen Blackmon^9^**: Neuropsychology Outcomes Core co‐Lead**; Kendi Muchungi^9^**: Neuroscientist, Post‐doctoral researcher for Ophthalmology module**; Laz U. Eze^2^**: Communications Core co‐Lead (Nigeria)**; Linda Khakali^9^**: Recruitment and community engagement strategist**; Manasi Kumar^14^**: Implementation Core co‐Lead**; Mansoor Saleh^15^**: Multi‐PI Nairobi, Kenya; **Miia Kivipelto^4‐6,16^**: Co‐Chief Investigator and co‐chair of the Africa‐FINGERS Executive Steering Group; **Muthoni Mwangi^8^**: Health Systems Implementation Core Investigator; **Njideka Okubadejo^17^**: Lagos‐Nigeria intervention trial co‐PI**; Rachel Maina^9^**: Trainee Neuropsychologist, Post‐doc; **Roselyter Rianga^8^**: Intervention Core Investigator; **Samuel Nguku^18^**: Molecular Imaging Core Co‐Lead and Study Clinician**; Shaheen Sayed^19^**: Biomarkers _Fluid and Neuropathology Core; **Sheena Shah^20^**: Biomarkers _Opthalmology Core; **Sheila Waa^18^**: Neuroradiologist, Study Clinician**; Sylvia Mbugua^9^**: Neurologist, Study Clinician**; Tamlyn Watermeyer^21^**: Neuropsychology Outcomes Core co‐Lead**; Thomas Karikari^22^**: Biomarkers _Fluid Core; **Thomas Thesen^9,23,^**: Biomarkers Imaging Core Co‐Lead; **Udunna Anazodo^24,25,26^**: Biomarkers Imaging Core Co‐Lead; **Valentine Ucheagwu^27^**: Eastern‐Nigeria intervention trial PI; **Executive Steering group members include**: Adesola Ogunniyi^28^, Agustin Ibanez^2,29^, Darina Bassil^30^, Francesca Mangialasche^4,5^, Henrik Zetterberg^21, 31,32,33,34,35,36^, Laura Baker^10^, Lucia Crivelli^12^, Lukoye Atwoli^9,37^, Michelle Mielke^10^, Olivera Nesic‐Taylor^9,38^, Ozioma C. Okonkwo^39^, Robert Perneczky^11,6,40,41,42,43^, Tunde Peto^13^, Victor Valcour^2,44^, Jennifer Yokoyama,^44^ and Zul Merali^9^.

^1^Department of Community Health and Primary care, University of Lagos, Oba Akinjobi Street, Ikeja, P.M.B. 21266, Lagos, Nigeria

^2^Global Brain Health Institute (GBHI) Atlantic Fellows for Equity in Brain Health; University of California, San Francisco (UCSF), 1651 4th St, 3rd Floor, San Francisco, CA 94158, USA; and Trinity College Institute of Neuroscience, Room 0.60 Lloyd Building, Trinity College Dublin, Dublin 2, D02 × 9W9, Ireland

^3^Institute for Advanced Medical Research and Training, College of Medicine, University of Ibadan, P.M.B 3017 G.P.O Ibadan, Oyo State, Nigeria

^4^Division of Clinical Geriatrics, Center for Alzheimer Research, Karolinska Institutet, Neo 7th floor, 141 83 Huddinge, Stockholm, Sweden

^5^FINGERS Brain Health Institute, c/o Stockholms Sjukhem Box 122 30, SE‐102 26, Stockholm, Sweden

^6^Ageing Epidemiology Research Unit, School of Public Health, Room 10L05, 10th Floor Lab Block, Imperial College London, Charing Cross Hospital, St Dunstan's Road, London W6 8RP, UK

^7^Department of Neurology, Institute of Clinical Medicine, University of Eastern Finland, Kuopio, P.O. Box 1627, Kuopio FI‐70211, Finland

^8^Department of Population Health, Aga Khan University, 3rd Parklands Avenue off Lumuru Road, P.O. Box 30270‐00100, Nairobi, Kenya

^9^Brain and Mind Institute, Aga Khan University, 3rd Parklands Avenue off Lumuru Road, P.O. Box 30270‐00100, Nairobi, Kenya

^10^School of Public Health Sciences, Wake Forest University, School of Medicine, Medical Center Boulevard, Winston‐Salem, NC 27157, USA

^11^Sheffield Institute for Translational Neuroscience, The University of Sheffield, 385a Glossop Rd, Broomhall, Sheffield S10 2HQ, UK

^12^Department of Cognitive Neurology, Fleni, Montañeses 2325, C1428 C1428AQK, Cdad. Autónoma de Buenos Aires, Argentina

^13^School of Medicine, Dentistry and Biomedical Sciences, Queen's University Belfast, 97 Lisburn Rd, Belfast BT9 7BL, UK

^14^Grossman School of Medicine, New York University, 550 1st Ave., New York, NY 10016, USA

^15^Cancer Research Unit, Aga Khan University, 3rd Parklands Avenue off Limiru Road, P.O. Box 30270‐00100, Nairobi, Kenya

^16^Institute of Public Health and Clinical Nutrition, University of Eastern Finland, P.O. Box 1627, FI‐70211 Kuopio, Finland

^17^Neurology Unit, Department of Medicine, College of Medicine, University of Lagos, University Road Lagos Mainland Akoka, Yaba, Lagos 101017, Nigeria

^18^Department of Radiology, Aga Khan University Hospital, 3rd Parklands Avenue off Lumuru Road, P.O. Box 30270‐00100, Nairobi, Kenya

^19^Department of Pathology, Aga Khan University, 3rd Parklands Avenue off Lumuru Road, P.O. Box 30270‐00100, Nairobi, Kenya

^20^Department of Ophthalmology, Aga Khan University, 3rd Parklands Avenue off Lumuru Road, P.O. Box 30270‐00100, Nairobi, Kenya

^21^Centre for Dementia Prevention, Clinical Brain Sciences, The University of Edinburgh, Outpatients Department, Level 2, Western General Hospital Crewe Road South, Edinburgh EH4 2XU, UK

^22^Department of Psychiatry, School of Medicine, University of Pittsburgh, Pittsburgh, PA 15213, USA

^23^Department of Medical Education, Geisel School of Medicine at Dartmouth, Hinman Box 7100, Remsen Building, Room 237, Hanover, NH 03755, USA

^24^Department of Neurology and Neurosurgery, Montreal Neurological Institute, McGill University, 3801 University Street, Irving Ludmer Building, 1033 Pine Avenue West, room 310, Montréal, Québec H3A 1A1, Canada

^25^Medical Artificial Intelligence Laboratory, Crestview Radiology, Ltd, J8M3+MGP, Old Otta Rd, Agege, Lagos 102212, Nigeria

^26^Department of Medicine (Level 6, Entrance 2, Old Main Building, J Floor) & Clinical & Radiation Oncology (L‐Block D‐Floor, Room D‐8), University of Cape Town, Groote Schuur Hospital, Observatory, Cape Town 7925, South Africa

^27^Department of Psychology, Nnamdi Azikiwe University, P.M.B. 5025, Awka, Anambra, Nigeria

^28^Institute for Advanced Medical Research and Training, College of Medicine, University of Ibadan, P.M.B. 3017 G.P.O. Ibadan, Oyo State, Nigeria

^29^Trinity College Institute for Neuroscience, Trinity College Dublin, Room 3.05, Lloyd Building, 42a Pearse St, Dublin D02 PX31, Ireland

^30^Harvard Center for Population and Development Studies, Harvard University, Cambridge, 9 Bow Street, Cambridge, MA 02138, USA

^31^Department of Psychiatry and Neurochemistry, Institute of Neuroscience and Physiology, the Sahlgrenska Academy at the University of Gothenburg, Medicinaregatan 11. 41390 Göteborg, P.O. Box 430, 40530 Göteborg, Mölndal, Sweden

^32^Clinical Neurochemistry Laboratory, Sahlgrenska University Hospital, Postcode SE‐43180, Mölndal, Sweden

^33^Department of Neurodegenerative Disease, UCL Institute of Neurology, Queen Square, London WC1N 3BG, UK

^34^UK Dementia Research Institute at UCL, 6th Floor, Maple House, Tottenham Ct Rd, Grower Street, London WC1E 6BT, UK

^35^Hong Kong Center for Neurodegenerative Diseases, Clear Water Bay, Hong Kong 518172, China

^36^Wisconsin Alzheimer's Disease Research Center, University of Wisconsin School of Medicine and Public Health, University of Wisconsin‐Madison, 600 Highland Avenue, MC 2420, Madison, WI 53792‐2420, USA

^37^Department of Medicine, Medical College East Africa, the Aga Khan University, 3rd Parklands Avenue off Lumuru Road, P.O. Box 30270‐00100, Nairobi, Kenya

^38^Tilman J. Fertitta Family College of Medicine, 5055 Medical Circle, Houston, TX 77204‐6064, USA

^39^Division of Geriatrics and Gerontology, Department of Medicine, University of Wisconsin, 2870 University Ave, Madison, WI 53705‐3611, USA

^40^Ludwig Maximilien University München, Nußbaumstr. 7, 80336 Munich, Germany

^41^Department of Psychiatry and Psychotherapy, LMU Hospital, Ludwig‐Maximilians‐Universität, Nußbaumstr. 7, München 80336, Germany

^42^German Center for Neurodegenerative Diseases (DZNE) Munich, Feodor‐Lynen‐Strasse 17, München, Bayern 81377, Germany

^43^Munich Cluster for Systems Neurology (SyNergy), Feodor‐Lynen‐Str. 17, 81377 Munich, Germany

^44^UCSF Weill Institute for Neurosciences, School of Medicine, University of California, 1651 4th Street, #371 M, San Francisco, CA 94158, USA
