# Supplementary material for: Dementia risk reduction in the African context: Multi‐national implementation of multimodal strategies to promote healthy brain aging in Africa (the Africa‐FINGERS project)
Source: Alzheimers Dement. 2024 Nov 7;20(12):8987–9003. doi: 10.1002/alz.14344 (PMC11667543; doi:10.1002/alz.14344)
Supplement: Supplementary file 2 — Supporting Information [file ALZ-20-8987-s001.docx]

**Dementia risk reduction in the African context: Multi-national implementation of multimodal strategies to promote healthy brain ageing in Africa (The AFRICA-FINGERS Project)**

**Appendix B**

**Prevalence of modifiable risk factors in a Nigerian Cohort of similar ethnic relationships to sub-populations of the Africa-FINGERS sample.**


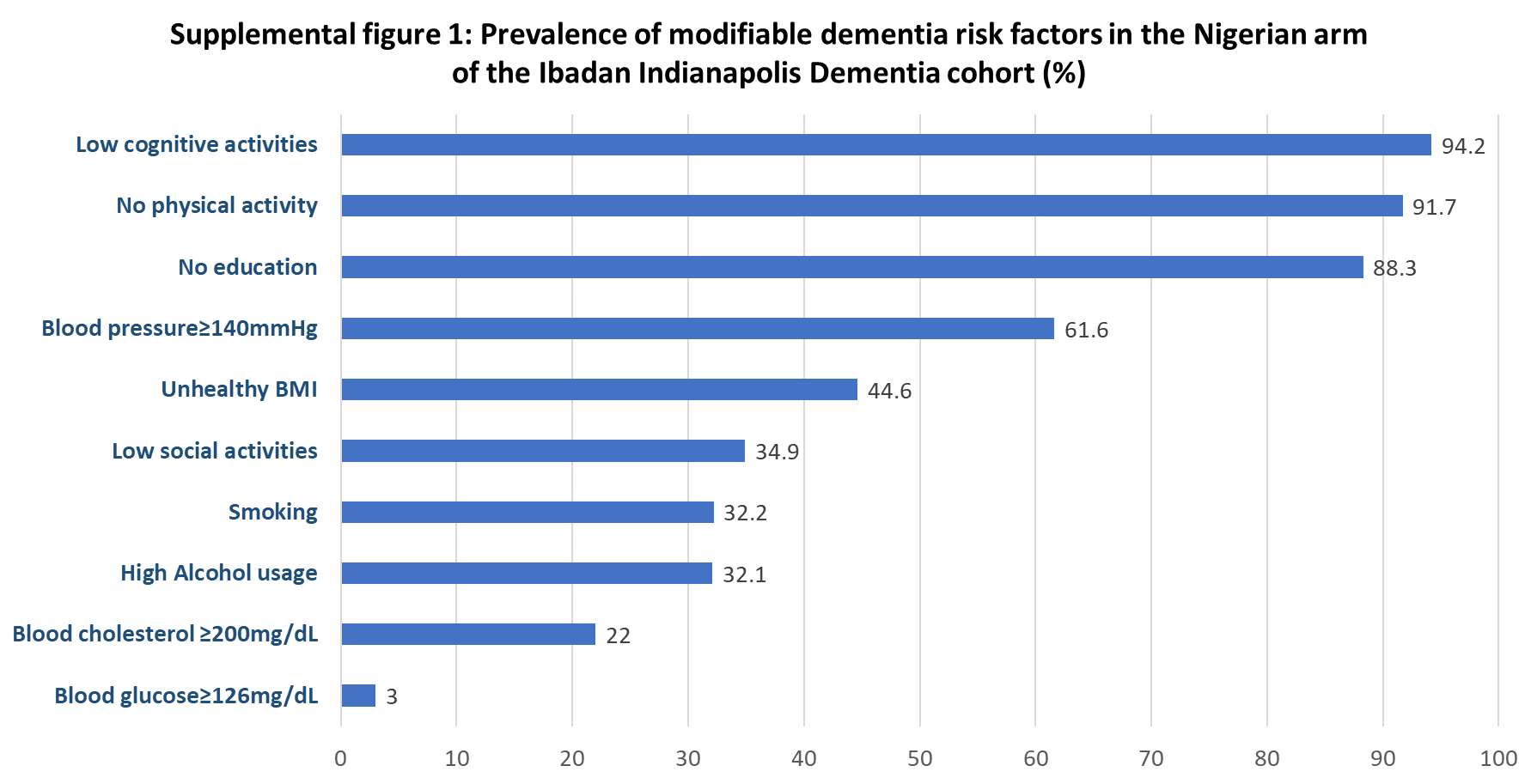


Our preliminary evaluation of dementia risk factors in the African context utilized longitudinal data from Nigeria (Indianapolis-Ibadan-Dementia study, 1992-2022, N=4353)^1^. Key modifiable risk factors identified included low levels of cognitive and social engagement, no education or physical activity, as well as high vascular risk factors. Low cognitive and physical activity as well as no education emerged with highest prevalence. (Supplemental Figure 2). Cox proportional hazard models showed that a vascular-based dementia risk score (CAIDE) without the APOE component predicted incident dementia (p<0.05).

**Appendix C**

**The African Dementia Imaging Protocol (ADIP) Development for Africa-FINGERS**

The African dementia imaging protocol (ADIP) is being designed for the Africa-FINGERS project with the intent for general use for African dementia research, where neuroimaging is indicated. The protocol provides a comprehensive image acquisition and analysis procedures for PET and MRI biomarkers relevant for dementia imaging in African populations. ADIP is based on the Alzheimer’s Disease Neuroimaging Initiative (ADNI) protocols (https://adni.loni.usc.edu/) and adapted to the imaging environments in Africa, which are predominately low-resourced and in clinical settings^2^. Each site participating in the Africa-FINGERS project will be qualified for MRI and PET imaging before the project starts using the ADNI qualification process. The scanners used will be approved following verification of phantom and human scan quality assurance (QA) checks as outlined in ADNI and the Canadian Dementia Imaging Protocol (CDIP) ^3, 4^. Upgrades to the scanner hardware (head coil) or software (version changes) during the study or amendments to ADIP acquisition components/parameters will require QA reassessment for approval.

Given the unique specifications of imaging systems across Africa, particularly MRI scanners and the lack of dementia imaging protocols adapted for imaging on these systems, the ADIP protocol will be systematically developed (supplementary figure 1) to ensure production of image qualities and biomarker metrics across sites and scanners that are consistent and compatible with established dementia studies (i.e., ADNI). The protocol consists of optimized standard imaging modalities for dementia research (core) and advanced imaging techniques relevant to assessing dementia risks in African populations (Table 1). An analysis pipeline for extracting established imaging biomarkers (volumetry, cortical thickness, cerebral blood flow (CBF), glucose uptake, etc.) will be developed and refined as the acquisition protocol is systematically developed. A validation study will be conducted to assess the performance of the optimized protocol and prescribe the safety and QA procedures for the Africa-FINGERS project. The final protocol will be reviewed by dementia imaging experts and approved for implementation in Africa-FINGERS project.

1. **Protocol Design Process**

The ADIP development process is briefly described below. The process considers the Africa-FINGERS project objectives, timeline, and deliverables.


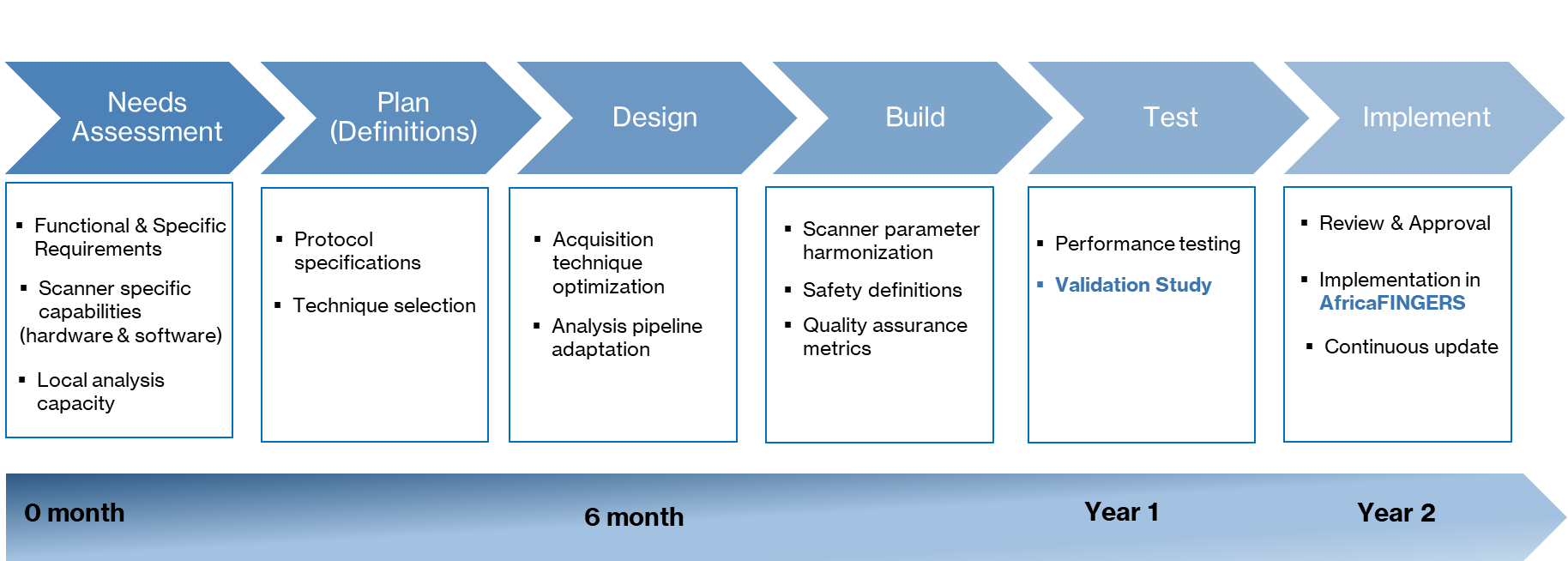
Supplementary Figure 2: The African Dementia Imaging Protocol design process

The site and scanner capacity assessments, protocol specifications, optimization, performance testing and validation studies will be conducted within the 1^st^ year of the 5-year AFRICA-FINGERS project and at the two study sites in Kenya and Nigeria. The proposed PET and MRI acquisition procedures outlined below will follow the scanner type and models at the project sites and updated with addition of new imaging systems, techniques, analysis methods or improvements in imaging capacities. The MRI validation scans will be acquired in 40 healthy controls (age 18 – 70 years old) at the Africa-FINGERS study sites using the optimized parameters designed and built onsite. Each site will scan 20 individuals twice to assess scanner/site-specific effects, reproducibility of biomarkers acquired and establish a harmonized standard condition for longitudinal imaging. Retrospective FDG-PET clinical scans will be used to evaluate the PET scanner performance and validation. Scan quality will be reviewed qualitatively by trained analysts to visually inspect subject motion, anatomical coverage, and presence of imaging artefacts^5^. The signal-to-noise (SNR) and contrast-to-noise (CNR) of anatomical MRI (T1-weighted and T2-weighted), perfusion MRI, and FDG-PET will be measured within gray and white matter using the CDIP approach^3^ and methods established by the group^,6^. QA will also include anatomical, functional, and diffusion MRI image quality metrics (IQMs) derived from the MRI Quality Control tool (MRIQC)^7^ using ADNI datasets as reference.

1. **Acquisition Protocol Guidelines**

The ADIP imaging modalities are summarized in the main manuscript (c.f. Table 1) with their alignment to ADNI, highlighted.

2.1 FDG-PET: Scans will be completed in Kenya on a General Electric (GE) PET/CT using the same procedure guidelines described in the ADNI-3 PET Technical Procedures. FDG-PET imaging will begin 30 minutes after intravenous injection of 185 MBq of FDG and data will be collected over 30 minutes.

2.2 MRI: 40–50-minute MRI scans will be acquired using sequence parameters adapted from ADNI-3 MRI protocols with similar scanner characteristics as the AFRICA-FINGERS sites in Kenya (Philips 3T) and Nigeria (Siemens 1.5T). The sequences include core anatomical T1-weighted (T1w) and T2-weighted (T2w), resting-state functional MRI (rsfMRI), arterial spin labelling (ASL), and diffusion weighted imaging (DWI). An advanced technique for regional exploration of physiological (i.e., cerebrovascular reactivity (CVR) brain outcomes will be implemented.

**References**:

1. Ogunniyi A, Baiyewu O, Gureje O, Hall KS, Unverzagt F, Siu SH, et al. Epidemiology of dementia in Nigeria: results from the Indianapolis-Ibadan study. Eur J Neurol. 2000;7(5):485-90.

2. Anazodo UC, Ng JJ, Ehiogu B, Obungoloch J, Fatade A, Mutsaerts H, et al. A framework for advancing sustainable magnetic resonance imaging access in Africa. NMR Biomed. 2023;36(3):e4846.

3. Duchesne S, Chouinard I, Potvin O, Fonov VS, Khademi A, Bartha R, et al. The Canadian Dementia Imaging Protocol: Harmonizing National Cohorts. J Magn Reson Imaging. 2019;49(2):456-65.

4. Jack CR, Jr., Bernstein MA, Fox NC, Thompson P, Alexander G, Harvey D, et al. The Alzheimer's Disease Neuroimaging Initiative (ADNI): MRI methods. J Magn Reson Imaging. 2008;27(4):685-91.

5. Anazodo UC, Finger E, Kwan BYM, Pavlosky W, Warrington JC, Günther M, et al. Using simultaneous PET/MRI to compare the accuracy of diagnosing frontotemporal dementia by arterial spin labelling MRI and FDG-PET. Neuroimage Clin. 2018;17:405-14.

6. Anazodo UC, Shoemaker JK, Suskin N, Ssali T, Wang DJ, St Lawrence KS. Impaired Cerebrovascular Function in Coronary Artery Disease Patients and Recovery Following Cardiac Rehabilitation. Front Aging Neurosci. 2015;7:224.

7. Esteban O, Birman D, Schaer M, Koyejo OO, Poldrack RA, Gorgolewski KJ. MRIQC: Advancing the automatic prediction of image quality in MRI from unseen sites. PLOS ONE. 2017;12(9):e0184661.
